# Targeted nanoparticle delivery of therapeutic antisense microRNAs presensitizes glioblastoma cells to lower effective doses of temozolomide *in vitro* and in a mouse model

**DOI:** 10.18632/oncotarget.25135

**Published:** 2018-04-20

**Authors:** Meenakshi Malhotra, Thillai Veerapazham Sekar, Jeyarama S. Ananta, Rammohan Devulapally, Rayhaneh Afjei, Husam A. Babikir, Ramasamy Paulmurugan, Tarik F. Massoud

**Affiliations:** ^1^ Laboratory for Experimental and Molecular Neuroimaging (LEMNI), Molecular Imaging Program at Stanford, Stanford University School of Medicine, Stanford, CA 94305, USA; ^2^ Cellular Pathway Imaging Laboratory (CPIL), Molecular Imaging Program at Stanford, Stanford University School of Medicine, Palo Alto, CA 94305, USA

**Keywords:** antagomiR, chemotherapy, microRNA-21, microRNA-10b, PLGA

## Abstract

Temozolomide (TMZ) chemotherapy for glioblastoma (GBM) is generally well tolerated at standard doses but it can cause side effects. GBMs overexpress microRNA-21 and microRNA-10b, two known oncomiRs that promote cancer development, progression and resistance to drug treatment. We hypothesized that systemic injection of antisense microRNAs (antagomiR-21 and antagomiR-10b) encapsulated in cRGD-tagged PEG-PLGA nanoparticles would result in high cellular delivery of intact functional antagomiRs, with consequent efficient therapeutic response and increased sensitivity of GBM cells to lower doses of TMZ. We synthesized both targeted and non-targeted nanoparticles, and characterized them for size, surface charge and encapsulation efficiency of antagomiRs. When using targeted nanoparticles in U87MG and Ln229 GBM cells, we showed higher uptake-associated improvement in sensitivity of these cells to lower concentrations of TMZ in medium. Co-inhibition of microRNA-21 and microRNA-10b reduced the number of viable cells and increased cell cycle arrest at G2/M phase upon TMZ treatment. We found a significant increase in expression of key target genes for microRNA-21 and microRNA-10b upon using targeted versus non-targeted nanoparticles. There was also significant reduction in tumor volume when using TMZ after pre-treatment with loaded nanoparticles in human GBM cell xenografts in mice. *In vivo* targeted nanoparticles plus different doses of TMZ showed a significant therapeutic response even at the lowest dose of TMZ, indicating that preloading cells with antagomiR-21 and antagomiR-10b increases cellular chemosensitivity towards lower TMZ doses. Future clinical applications of this combination therapy may result in improved GBM response by using lower doses of TMZ and reducing nonspecific treatment side effects.

## INTRODUCTION

Glioblastoma (GBM) is the most malignant of primary brain tumors [1] and the twelfth leading cause of cancer-related deaths in the United States [2]. Conventional treatment methods include surgery, radiation therapy and chemotherapy. However, despite this multimodal therapeutic approach the median survival rate of patients remains at 14.6 months after diagnosis [3, 4], made worse by the high rate of relapse after surgery [5, 6]. Several factors, such as resistance to conventional chemoradiation and differential response rates of heterogeneous cancer cell populations within tumors limit GBM treatment [7]. The most commonly used adjuvant chemotherapeutic drug for GBM is temozolomide (TMZ). It is a prodrug of the alkylating agent 5-(3 methyltriazen-1-yl) imidazole-4-carboximide (MTIC), which disrupts DNA replication and causes programmed cell death (apoptosis) in rapidly dividing cells [8, 9]. Although TMZ is generally well tolerated by patients, it is however associated with mild to moderate side effects such as fatigue, nausea, vomiting, thrombocytopenia, and neutropenia. In some cases, severe hematologic adverse events, including myelodysplastic syndrome and aplastic anemia have been reported, which are mainly due to the high doses of TMZ prescribed for treatment [8, 9].

MicroRNAs (miRNAs or miRs) are small (18-22 nucleotides) non-coding RNAs that regulate gene expression by directly binding to target messenger RNAs (mRNAs), resulting in mRNA degradation or translational repression. By negatively regulating their target mRNAs, miRNAs can act either as tumor suppressors or oncogenes (oncomiRs) [10]. Indeed, dysregulated miRNA expression is commonly reported in various human cancers including GBM. In general, altering the expression of miRNAs has significant implications on cell viability as well as strategies to overcome cancer cell resistance to chemotherapeutic drugs [10]. Hence, miRNA-targeted treatments are emerging as a promising new generation of molecular therapeutic strategies for cancer, including GBM. In contrast to the use of standard chemotherapeutics, a more targeted and personalized GBM treatment, such as by regulating the expression of genes associated with cancer progression and drug resistance using therapeutic miRNAs (that yield global pathway regulation, as opposed to RNA interference (RNAi) strategies, where a single gene is targeted), may provide potentially useful and biologically more meaningful avenues to treat GBM with less side-effects [10].

The expression levels of miRNAs in cancer cells can predict poor survival, rapid proliferation, metastasis, and treatment resistance [10]. Specifically, miR-21 has been identified as a potent oncomiR overexpressed in the majority of GBMs [10]. A recent study identified the tumor suppressor insulin-like growth factor binding protein-3 (IGFBP-3) as one of the targets of miR-21 [11]. Other associated targets of miR-21 include RECK, TIMP3, APAF1, ANP32A, SMARCA4, Caspases, PTEN, Cdc25A, HNRPK, TAp63, Spry2, LRRFIP1, and PDCD4 [12, 13]. Another microRNA, miR-10b is also overexpressed in GBM and increases the invasive capabilities of these high-grade tumors. MiR-10b has been found to regulate the expression of RhoC and uPAR via targeting the transcription factor HoxD10 [14]. Thus, targeting miR-21 and miR-10b using antisense microRNAs (antagomiRs) may represent a useful anticancer molecular therapy for GBM. Indeed, inhibition of miR-21 and miR-10b has also been found to induce cell cycle arrest and reduce migration and apoptosis [15, 16], and enhance response of GBM cells to TMZ [17, 18].

Synthetic naked miRNAs rapidly degrade in plasma. In order to improve the delivery of synthetic sense and antisense miRNAs *in vivo*, and to reduce their degradation in the systemic circulation, various delivery systems have been proposed with the potential for clinical translation. Nanoparticles, with their unique size, shape and surface properties, are under intense scrutiny as potential drug and gene delivery platforms to the brain. The preferential accumulation of some nanoparticles in tumors and their ability to encapsulate and deliver therapeutic molecules in a ‘Trojan horse’ fashion makes them attractive candidates for miRNA delivery to GBMs. In addition, the external surface of nanoparticles can be functionalized with targeting peptides or antibodies to enhance selective delivery of miRNAs to GBM cells. Previous studies have explored the potential of non-targeted Poly(lactic-co-glycolic acid) (PLGA) nanoparticles to deliver antagomiR-21 and antagomiR-10b to GBM cells in culture [15], demonstrating enhanced chemosensitivity of GBM cells towards TMZ. Here, we investigate whether GBM cellular uptake can be improved by using cRGD-targeted PLGA-PEG nanoparticles encapsulating antagomiR-21 and antagomiR-10b both in cell culture and *in vivo*. cRGD binds with high affinity to α_v_β_3_ integrin receptors on angiogenic blood vessels and cancer cell surfaces [19]. By extension, we also evaluate the strategy of sensitizing GBM cells (using delivered antagomiRs) prior to TMZ treatment, and study the effects of both antagomiRs and TMZ on cellular proliferation, apoptosis, and inhibition of endogenous miRNAs function and their downstream effects on tumor suppressor and apoptotic gene (PTEN, PDCD4, HOXD10, P53 and CASP3) expression. Our results demonstrate enhanced cellular uptake of cRGD-targeted nanoparticles carrying therapeutic antagomiR-21 and antagomiR-10b, and the effectiveness of this strategy in lowering the dosage of concurrent TMZ to reduce tumor volumes for GBM cell xenografts in mice.

## RESULTS

### Synthesis, preparation, and characterization of cRGD-targeted and non-targeted PLGA nanoparticles encapsulating antagomiR-21 and antagomiR-10b

**Figure 1:**
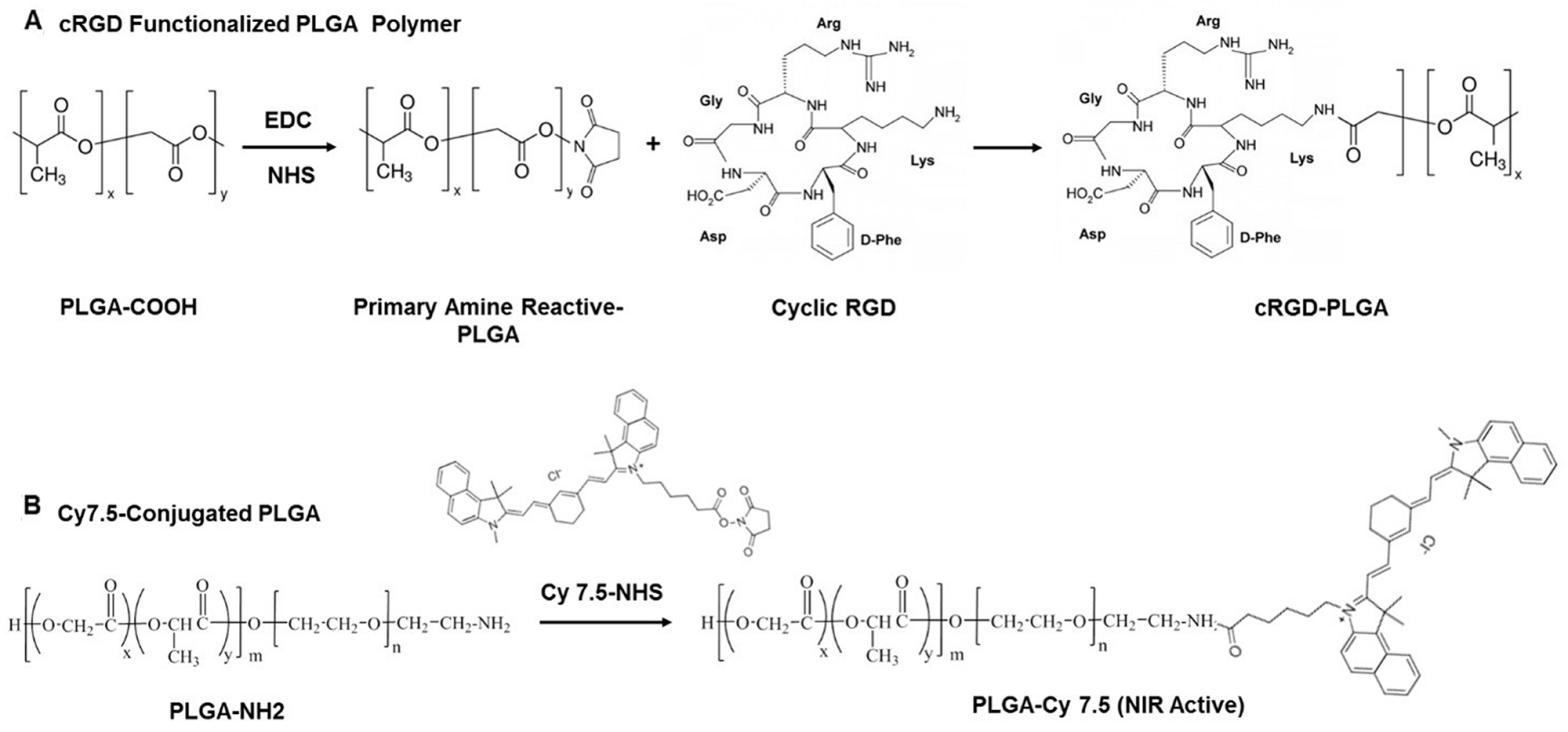
Schematic representing synthesis of **(A)** cRGD-functionalized PLGA polymer, and **(B)** Cy7.5-conjugated PLGA polymer.

**Figure 2:**
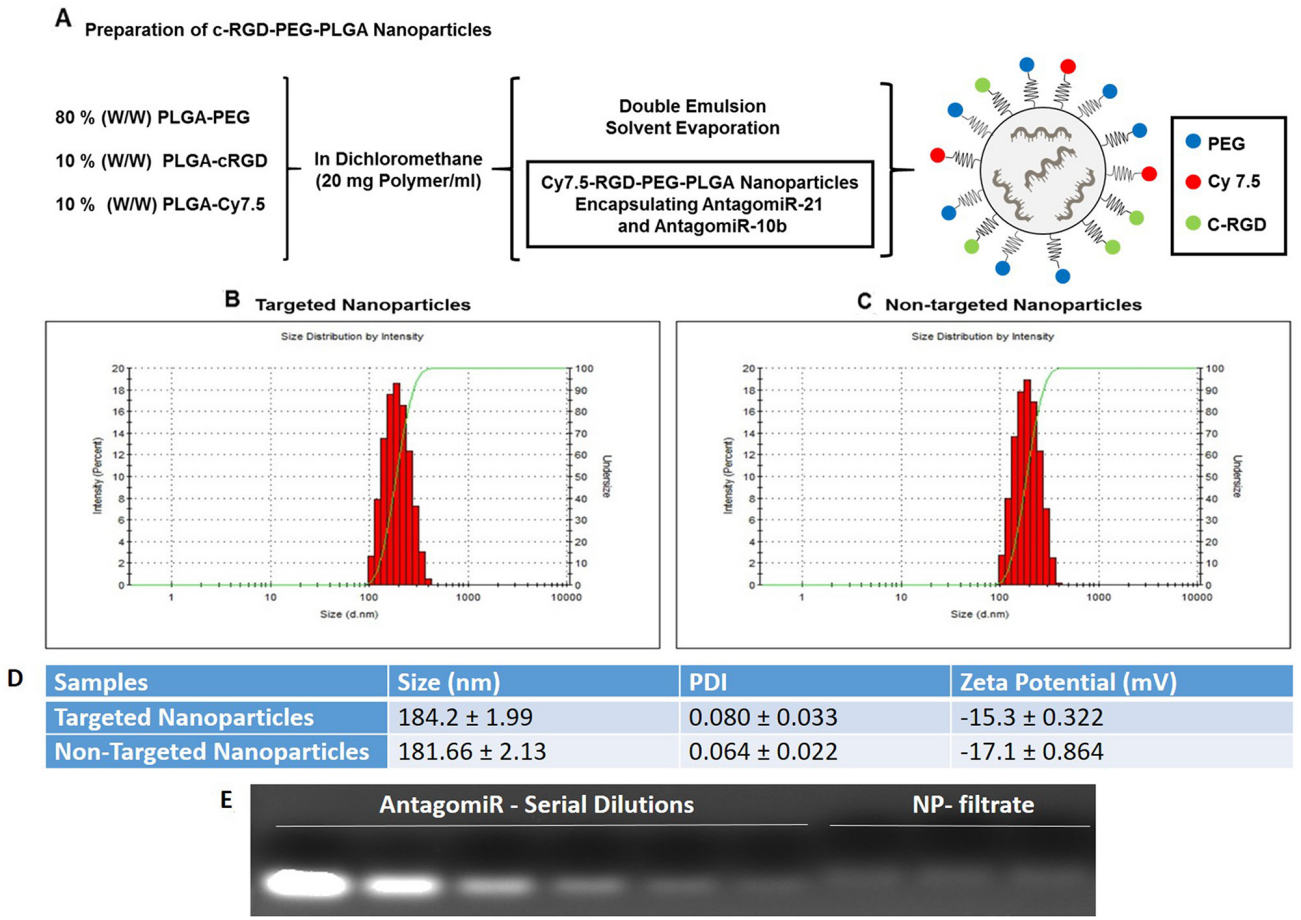
**(A)** Synthesis scheme and diagram of cRGD-targeted nanoparticle following a double emulsion solvent evaporation method. **(B and C)** Representative DLS measurement for the distribution of cRGD-targeted and non-targeted nanoparticles. **(D)** Table showing the size, polydispersity index and zeta potential of antagomiR-21 and antagomiR-10b co-loaded cRGD-targeted and non-targeted nanoparticles. **(E)** Agarose gel retardation assay to determine the amount of loaded antagomiR-21 and antagomiR-10b in cRGD-targeted nanoparticle in filtrate obtained after purification of nanoparticle fraction

We prepared cRGD-functionalized PLGA polymer and Cy7.5-conjugated PLGA polymer as shown schematically in Figure 1A and 1B. We prepared cRGD-targeted and non-targeted nanoparticles following a double-emulsion solvent evaporation method described previously [15, 17]. Figure 2A shows the schematic of developing cRGD-targeted nanoparticles comprising of PLGA-PEG: PLGA-cRGD: PLGA-Cy7.5 in the ratio 80:10:10. We confirmed the morphology of the nanoparticles by transmission electron microscopy (TEM) after staining with 1% phosphotungstic acid. The non-targeted nanoparticles comprised PLGA-PEG: PLGA-Cy7.5 in the ratio 90:10. We analyzed the targeted and non-targeted nanoparticles for size distribution and zeta-potential by dynamic light scattering (Figure 2B and 2C) and found the size to be of 184.2 ± 1.99 nm and 181.66 ± 2.13 nm, with polydispersity index of 0.080 ± 0.033 and 0.064 ± 0.022, respectively. We obtained zeta potential of −15.3 ± 0.322 and −17.1 ± 0.864, for targeted and non-targeted nanoparticles, respectively (Figure 2D). We achieved successful encapsulation of antagomiRs at >90% efficiency (Figure 2E). Specifically, we developed a standard curve of antagomiRs with serial dilutions, and the amount of free/unloaded antagomiR in the nanoparticle filtrate was quantified based on the standard curve. Previous studies had determined that co-loading of antagomiR-21 and antagomiR-10b was of nearly equimolar concentrations, after analyzing nanoparticles prepared in different batches [17].

### Cellular uptake of cRGD-targeted and non-targeted PLGA nanoparticles encapsulating antagomiR-21 and antagomiR-10b in U87MG and Ln229 GBM cells

**Figure 3:**
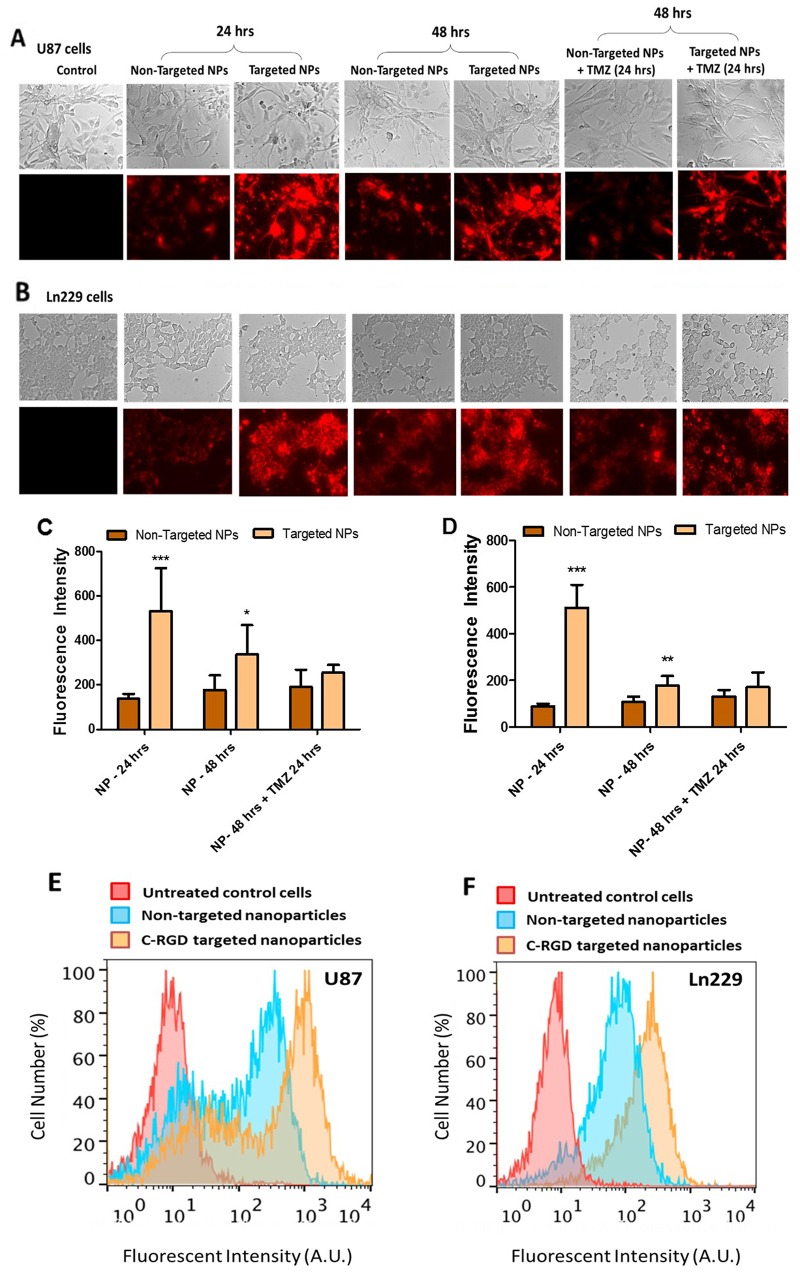
*In vitro* cell uptake analysis of cRGD-targeted PEG-PLGA nanoparticles compared to non-targeted PEG-PLGA nanoparticles in U87MG and Ln229 cells The nanoparticles were prepared with 10% Cy7.5-conjugated PLGA polymer. **(A and B)** represent the fluorescence image (magnification ×20), indicative of cellular uptake of nanoparticles. **(C and D)** Quantitative analysis of cellular uptake in U87MG and Ln229 cells, respectively, using Image J (n=5). The data are presented as mean ± SEM; ^*^ represents *P* ≤ 0.05, ^**^ represents *P* ≤ 0.01 and ^***^ represents *P* ≤ 0.001. **(E and F)** Flow cytometry (FACS) analysis of cellular uptake of nanoparticles in U87MG and Ln229 cells.

We performed cellular uptake analysis with cRGD-targeted and non-targeted nanoparticles in U87MG and Ln229 GBM cells. The presence of Cy7.5 in PLGA polymer nanoparticles facilitated visualization and quantitation of the percentage of nanoparticle uptake by fluorescent spectroscopic analysis. Cellular uptake of nanoparticles occurs either by adsorption and passive diffusion across cell membrane or by interaction with a receptor prior to receptor-mediated endocytosis in the case of ligand-conjugated nanoparticles [20]. In our study, the nanoparticles were PEGylated and carried a negative surface charge. This ensured that the nanoparticle cellular uptake would majorly depend on or be enhanced by the targeting ligand (cRGD) present on the surface of nanoparticles. We observed a significant difference in the fluorescence intensity between cells treated with cRGD-targeted and non-targeted nanoparticles in both U87MG (Figure 3A and 3C) and Ln229 (Figure 3B and 3D) cells. We found a maximum difference between the uptake of cRGD-targeted nanoparticles compared to non-targeted nanoparticles (~3-fold) at 24 h post treatment. After 48 h, no significant difference in the uptake of nanoparticles from cells treated with either non-targeted or cRGD-targeted nanoparticles was observed. We speculate that this is because continuous treatment of cells with nanoparticles may also permit their continued passive uptake to some extent, thus increasing intracellular levels of nanoparticles over time. In addition, treatment of cells by nanoparticles loaded with antagoimiR-10b or anitagomiR-21, as well as TMZ, enhances cellular response to treatment, which may alter the membrane potential of cells [21] to further enhance fluorescence signal, independent of receptor mediated endocytosis. We also measured cellular uptake of Cy7.5-labeled targeted and non-targeted nanoparticles in U87MG and Ln229 cells by FACS analysis 24 h after treatment, and found significantly increased fluorescence signal by targeted nanoparticles compared to non-targeted ones in both cells (Figure 3E-3F).

### Cell viability assay evaluates the effectiveness of delivered cRGD-targeted and non-targeted PLGA nanoparticles encapsulating antagomiR-21 and antagomiR-10b to pre-sensitize U87MG and Ln229 GBM cells to TMZ treatment

**Figure 4:**
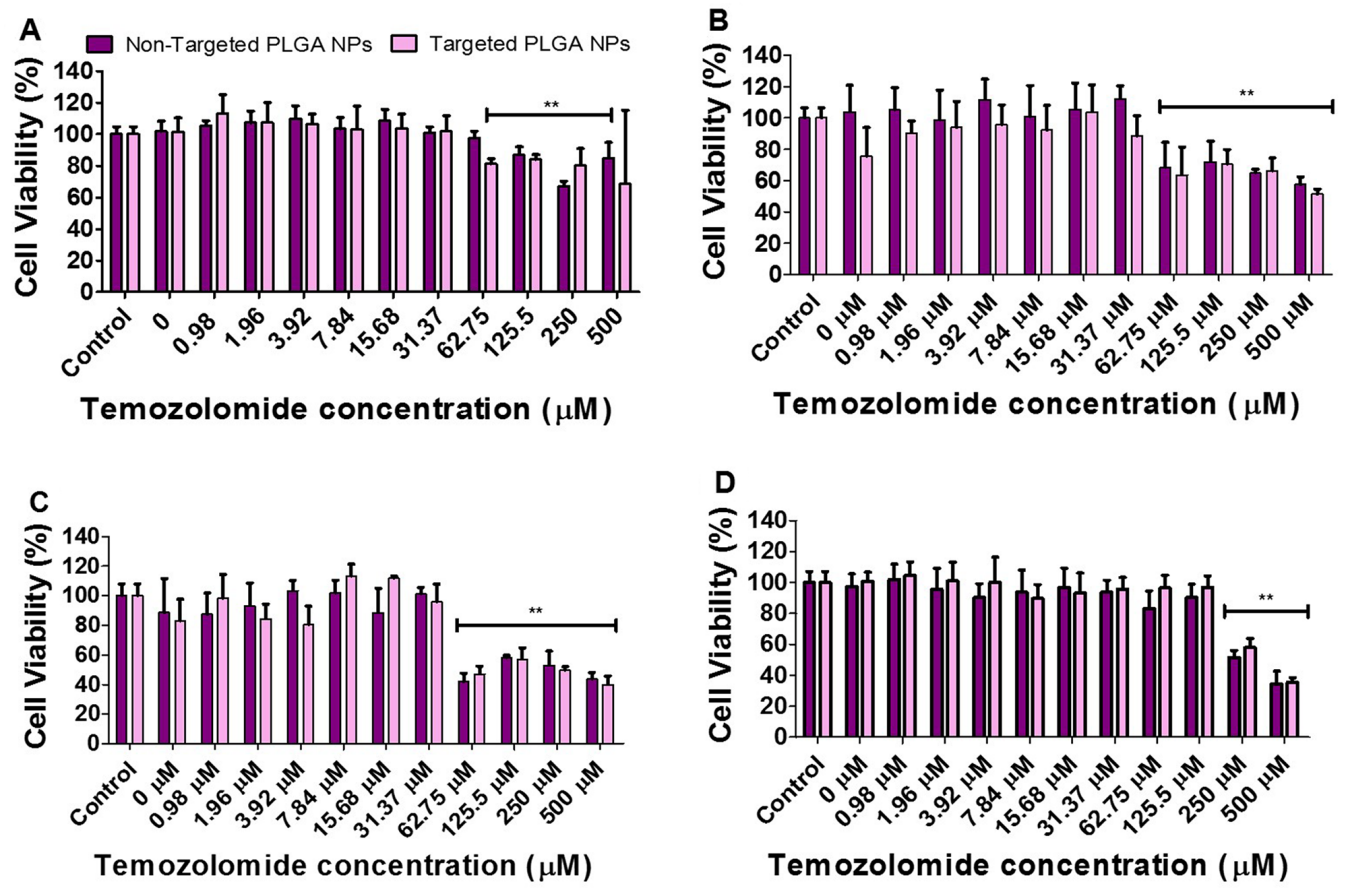
Cell viability analysis performed on: U87MG cells **(A and B)** and Ln229 cells **(C and D)** at 24 h and 48 h, respectively. The cells were treated with cRGD-targeted and non-targeted PLGA nanoparticles carrying 10 pmoles of each antagomiR-21 and antagomiR-10b, post-treated with different concentrations of TMZ. The data is presented as mean ± SEM; ^*^ represents *P* ≤ 0.05, ^**^ represents *P* ≤ 0.01.

We evaluated the antiproliferative and cytotoxic effects of cRGD-targeted and non-targeted PLGA nanoparticles co-delivering antagomiR-21 and antagomiR-10b (10 pmoles each), along with increasing concentrations of TMZ (0 to 500 μM) treatment on U87MG and Ln229 cells. We pre-treated the cells with nanoparticles for 24 h prior to TMZ treatment, and evaluated the cytotoxicity at 24 h and 48 h post TMZ treatment. Figure 4 represents cell viability data at 24 h and 48 h for U87MG cells (Figure 4A, 4B) and Ln229 cells (Figure 4C, 4D). We observed a significant reduction (*P* < 0.01) in cell viability at a TMZ concentration of 62.75 μM and above, at 24 h and 48 h for U87MG cells, and at 24 h but not at 48 h for Ln229 cells. We speculate that, unlike U87MG cells, Ln229 cells have mutant p53 and they therefore possess a compromised apoptotic pathway that facilitates cell survival and recovery from drug response when no further active prodrug (i.e. TMZ) conversion occurs to stress the cells towards death. Thus, the observed difference in cell viability results for Ln229 cells at 24 h and 48 h is considerably influenced by the dynamics of its growth cycle and the stability of TMZ in the medium. It was also evident from this study that cRGD-targeted and non-targeted nanoparticles were non-toxic to cells. Moreover, antagomiR-10b and antagomiR-21 only show cytostatic effects while enhancing cell response to chemotherapy rather than killing the cells.

### FACS analysis measures induced apoptosis and cell cycle status of U87MG and Ln229 GBM cells pre-treated with PLGA nanoparticles encapsulating antagomiR-21 plus antagomiR-10b and co-treated with TMZ

**Figure 5:**
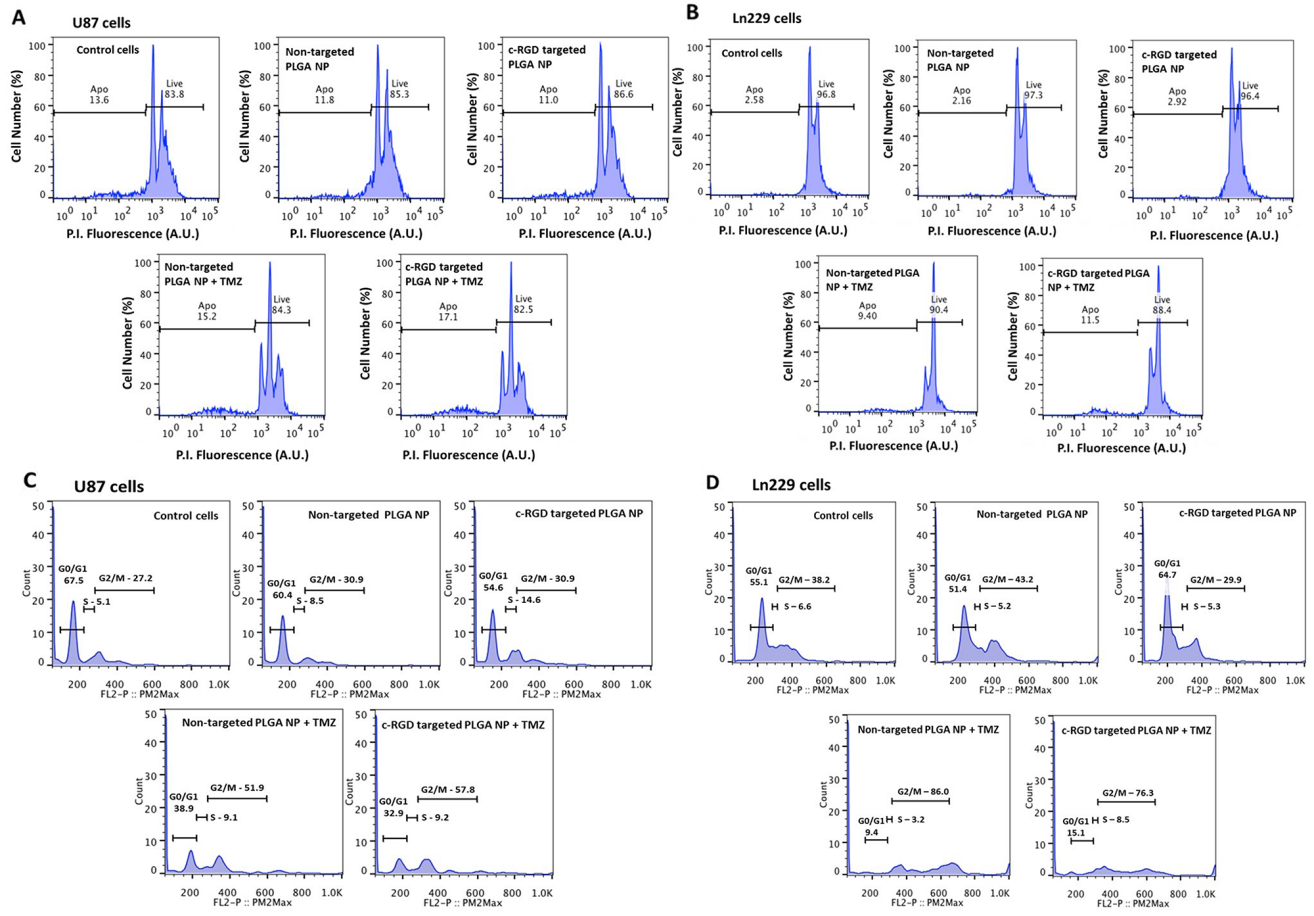
Flow cytometry analysis (FACS) of cells stained with propidium iodide for **(A and B)** live and dead cells and **(C and D)** cell cycle analysis, representing percentage distribution of cells at different phases of cell cycle. The cells were treated with cRGD-targeted and non-targeted PLGA nanoparticles, with and without post-treatment with 500 μM TMZ.

We performed flow cytometry analysis to evaluate cellular apoptosis (live/dead cell assay), and cell cycle status after different treatment conditions using propidium iodide as a cell staining dye (based on their DNA content, DNA-fragment distribution and nuclear architecture). As shown in Figure 5A (U87MG cells) and Figure 5B (Ln229 cells), there was no significant difference between the apoptotic populations in cells treated with either cRGD-targeted or non-targeted PLGA nanoparticles co-delivering antagomiR-21 and antagomiR-10b, when compared with untreated control cells. However, upon co-treatment with TMZ the number of apoptotic cells increased significantly from both cells treated with cRGD-targeted and non-targeted PLGA nanoparticles encapsulating antagomiR-21 and antagomiR-10b, compared to control cells. Specifically, cells treated with cRGD-targeted nanoparticles and then post-treated with TMZ showed increased apoptosis (17.1% and 11.5% of FACS gated U87MG and Ln229 cells, respectively) when compared with the non-targeted nanoparticles, and post-treated with TMZ (15.2% and 11.5%, respectively). We also analyzed the effects of treatment on different cell cycle phases. A previous study had indicated that co-delivery of antagomiR-21 and antagomiR-10b, followed by TMZ treatment, causes cell cycle arrest at G2/M phase [15]. In addition, it was observed that cells transfected with antagomiR-21 and antagomiR-10b alone or together, without TMZ treatment, do not cause any significant accumulation at G2/M phase. Further, TMZ alone is majorly responsible for the increase in G2/M cell accumulation compared to untreated control cells. The results in Figure 5C and 5D showed a significant increase in the G2/M phase of the cell cycle only after treatment with TMZ (G2/M phase: 51.9% for non-targeted, and 57.8% for cRGD-targeted nanoparticles [for U87MG cells]; 86.0% for non-targeted and 76.3% for cRGD-targeted nanoparticles [for Ln229 cells]) when compared with the untreated control (27.2% for U87MG cells, and 38.2% for Ln229 cells). These findings concurred with prior results [15], and indicated that TMZ was majorly responsible for inducing cell cycle arrest and inhibiting cell proliferation in U87MG and Ln229 cells.

### qRT-PCR analysis detects the modulation of downstream target genes of miR-21 and miR-10b expression in cells treated with PLGA-PEG nanoparticles

**Figure 6:**
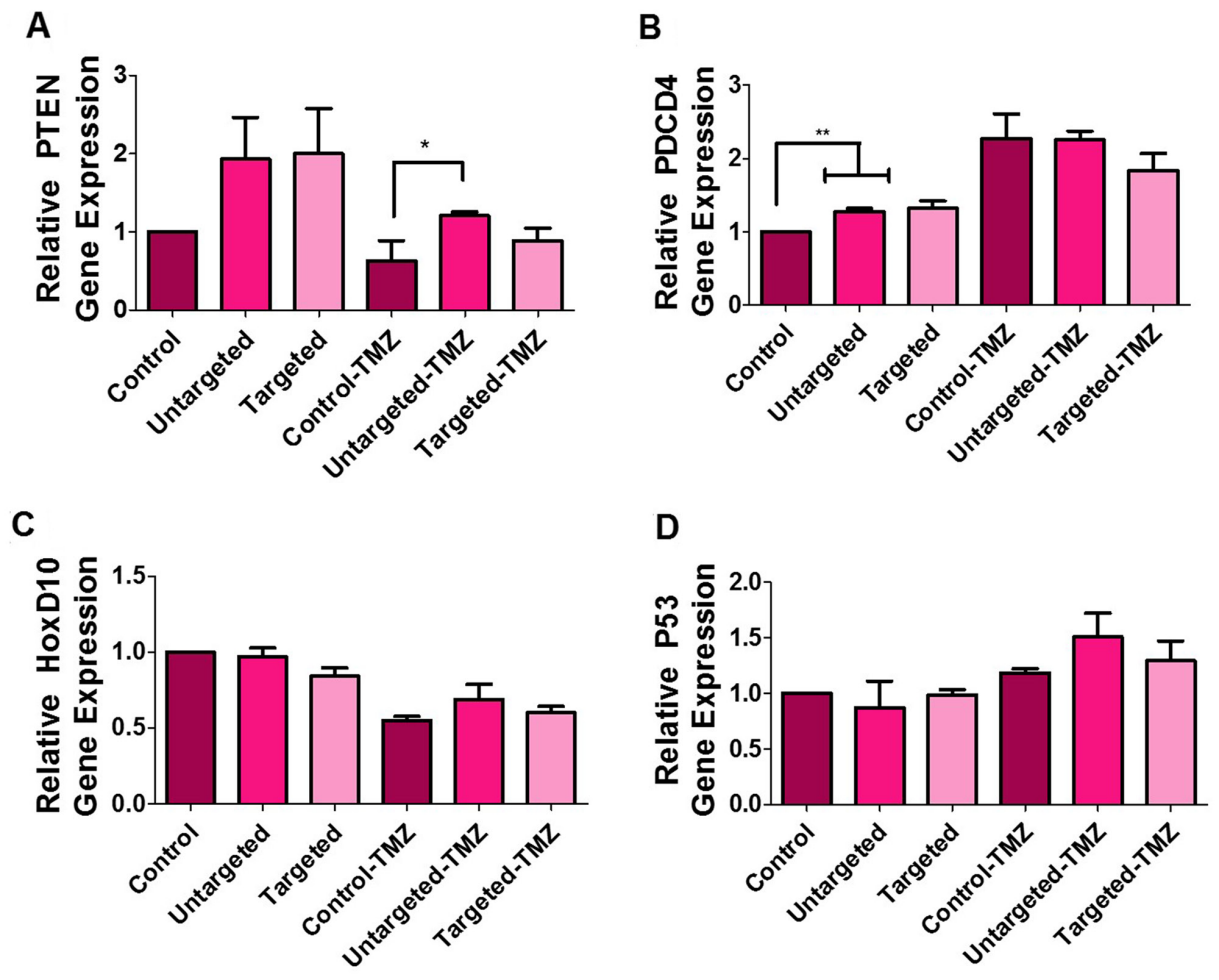
Quantitative real-time PCR analysis of U87MG cells The graph represents relative gene expression of **(A)** PTEN, **(B)** PDCD4, **(C)** HOXD10 and **(D)** P53 in cells treated with untargeted and targeted nanoparticles, with and without TMZ (500 μM) treatment. The data is presented as mean ± SD; ^*^ represents *P* ≤ 0.05, ^**^ represents *P* ≤ 0.01 and ^***^ represents *P* ≤ 0.001.

We evaluated the mRNA expression of PTEN, PDCD4, HOXD10, and p53 as indicators of miR-21 and miR-10b suppression in U87MG cells after co-delivering antagomiR-21 and antagomiR-10b using cRGD-targeted and non-targeted PLGA-PEG nanoparticles, with and without TMZ treatment. In previous studies, co-treating U87MG cells with antagomiR-21 and antagomiR-10b had led to an upregulation of the tumor suppressor genes PTEN and PDCD4 [15]. Here, we observed a higher expression of PTEN in cells treated with targeted and non-targeted nanoparticles, when compared with the control cells. Interestingly, the levels of PTEN dropped upon treatment of cells with TMZ, likely owing to the domination of the cytostatic effect caused by TMZ, compared to PTEN-mediated initiation of the apoptotic pathway. However, the cells treated with nanoparticles, followed by TMZ treatment, showed significantly (*P* < 0.05) higher PTEN levels when compared with their respective controls (Figure 6A). We observed a significantly higher (*P* < 0.01) relative expression of PDCD4 in nanoparticle-treated cells. Although PDCD4 expression after TMZ treatment was higher when compared with samples without TMZ treatment, we found no significant differences among control, non-targeted, and targeted nanoparticles for cells treated with TMZ (Figure 6B). HoxD10 is an important indicator of miR-10b suppression. The treatment of cells with non-targeted and targeted nanoparticles, regardless of TMZ treatment, showed no significant difference with their respective control (Figure 6C). Similar to previous findings, we attributed the lack of difference in HOXD10 expression to the likely low endogenous levels of miR-10b in U87MG cells (500 to 1,500 copies/cell), as compared to miR-21 (60,000 copies/cell) [15]. P53 is known to be a target for miR-21 and miR-10b [22, 23]. We found increased levels of p53 in cells treated with both non-targeted and targeted nanoparticles only after TMZ treatment (Figure 6D).

### Immunoblot analysis detects the modulation of downstream target genes of miR-21 and miR-10b expression in cells treated with PLGA-PEG nanoparticles

**Figure 7:**
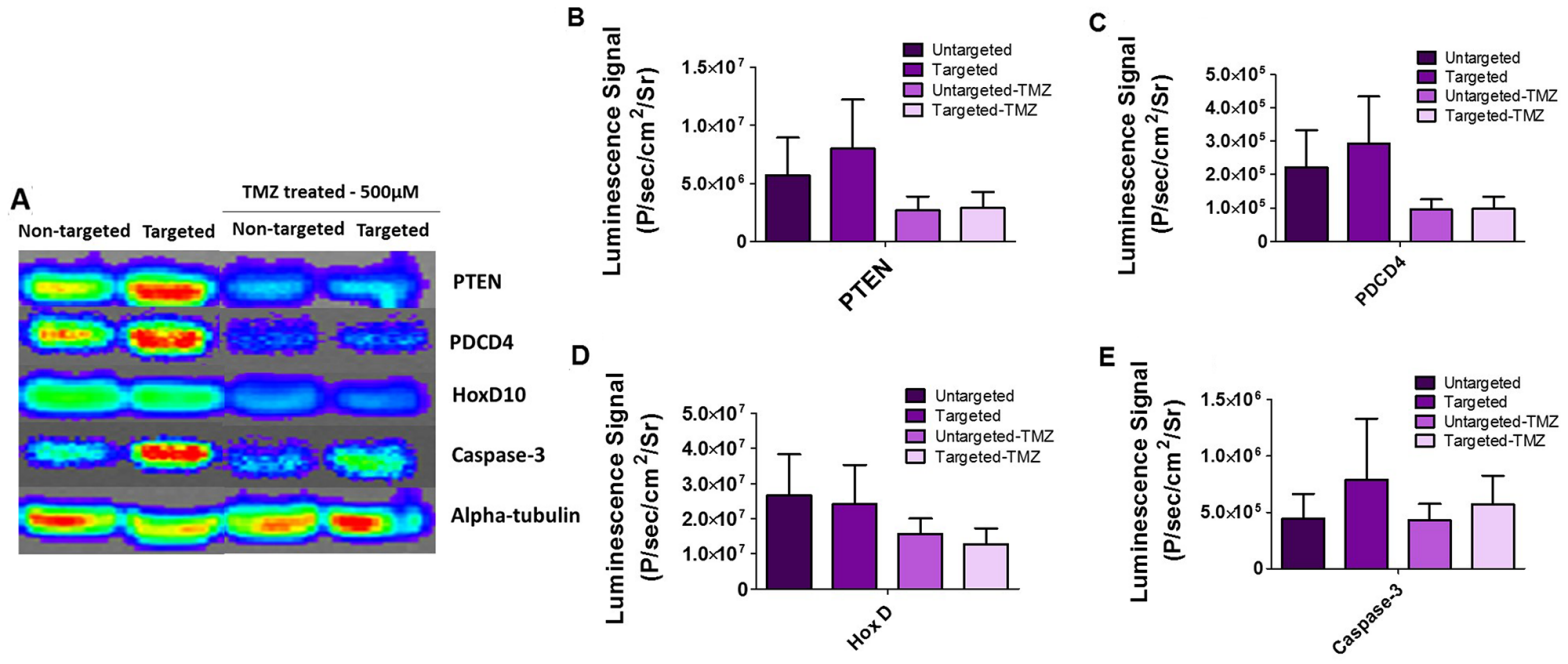
Immunoblot analysis of U87MG cells treated with cRGD-targeted PEG-PLGA nanoparticles and non-targeted PEG-PLGA nanoparticles, with and without TMZ treatment **(A)** The blot was stained with respective antibodies for PTEN, PDCD4, HOXD10, Caspase-3, alpha-tubulin and imaged by optical CCD camera. **(B, C, D and E)** Quantitative representation of PTEN, PDCD4, HOX-D and Caspase 3 on an immunoblot by measuring the average luminescence radiance using IVIS live imaging software.

Using immunoblot analysis, we further validated the expression of key targets observed by qRT-PCR analysis. Thus, we analyzed the key targets for miR-21, i.e. PTEN, and PDCD4; and the key target for miR-10b, i.e. HOXD10 and other apoptotic targets such as Caspase-3, all to confirm translational silencing when using antagomiR-21 and antagomiR-10b delivered through targeted and non-targeted nanoparticles, with and without TMZ treatment. As shown in Figure 7, we observed high expression of PTEN, PDCD4 and CASP3 (but not HOXD10) in cells treated with targeted nanoparticles when compared to the non-targeted nanoparticles. There was no significant difference in HOXD10 expression, likely owing to the low copy number of miR-10b in U87MG cells [15]. Similarly, the change in HOXD10 expression was not significant when compared to the expression of other target genes (PTEN and PDCD4) for miR-21, which has a high copy number in U87MG cells. The cells treated with high dose (500 μM) TMZ showed decreased expression of target proteins as expected because of increased cell death.

### Systemically delivered cRGD-targeted PLGA-PEG nanoparticles encapsulating antagomiR-21 and antagomiR-10b followed by intraperitoneal administration of TMZ showed significant levels of antitumor effect in mice

**Figure 8:**
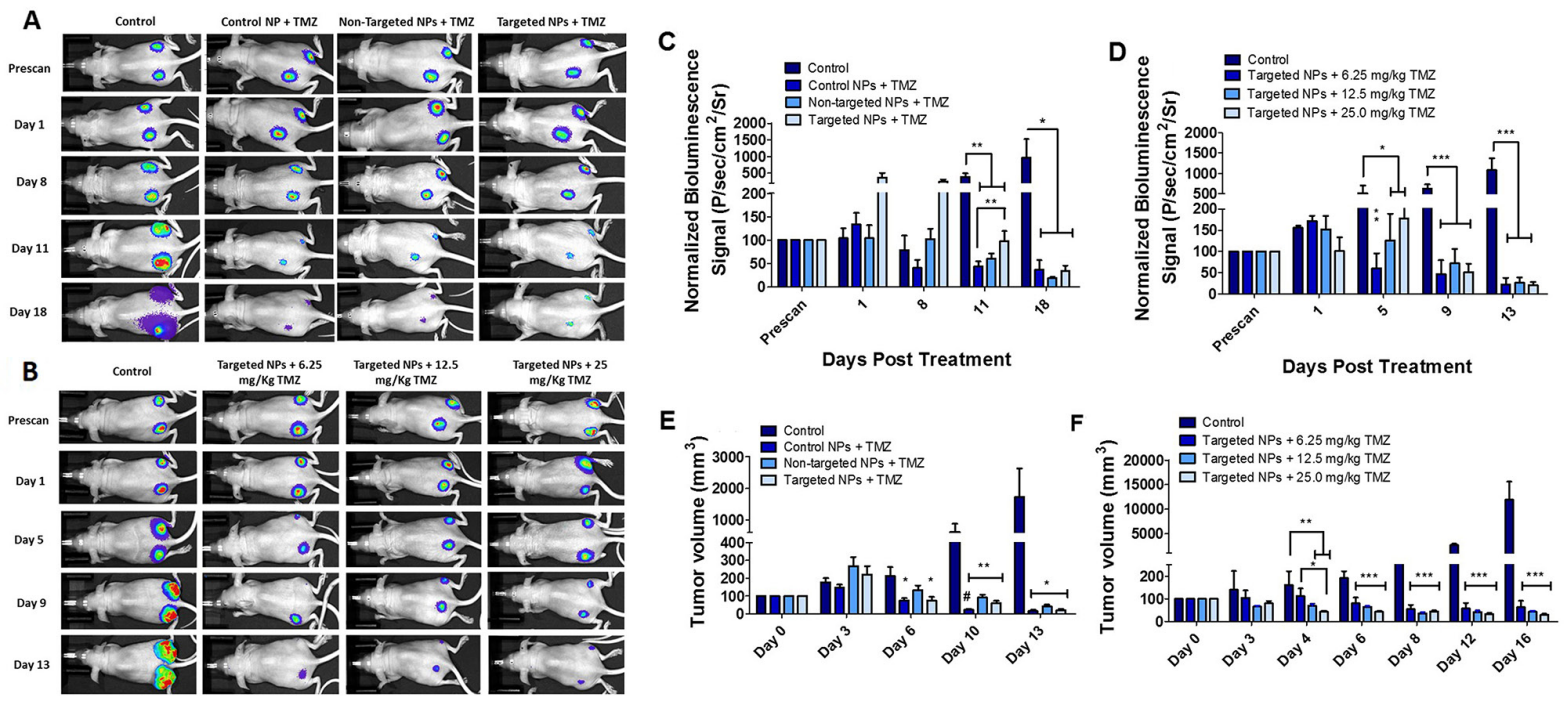
*In vivo* tumor growth analysis and bioluminescence imaging of mice bearing U87MG tumors stably expressing Fluc-eGFP The batch ‘A’ data are represented in **(A)**, **(C)** and **(E)**, wherein the animals were treated with Control (unmodified) PLGA, non-targeted PEG-PLGA and cRGD-targeted PEG-PLGA nanoparticles carrying 280 pmoles of antagomiR-21 and antagomiR-10b each. The animals were also treated with 25 mg/kg (body weight) of TMZ. The batch ‘B’ data are represented in **(B)**, **(D)** and **(F)**, wherein the animals were treated with cRGD-targeted PEG-PLGA nanoparticles carrying 280 pmoles of antagomiR-21 and antagomiR-10b each. (A and B) Optical bioluminescence imaging of mice bearing U87MG tumors. (C and D). Quantification of bioluminescence signal from animals shown in (A) and (B), respectively. (E and F) Tumor growth volume (mm^3^) measured in different treatment groups over time. The animals were treated with different doses of TMZ. The data are presented as mean ± SEM; ^*^ represents *P* ≤ 0.05, ^**^ represents *P* ≤ 0.01, ^***^ represents *P* ≤ 0.001, ^#^ represents *P* ≤ 0.001 between control nanoparticles and cRGD-targeted PEG-PLGA nanoparticles.

Figure 8 presents the data from animal experiments where we quantified the optical bioluminescence imaging of mice bearing tumor xenografts of GBM cells. In the first cohort (batch ‘A’), mice received a fixed dose of TMZ after antimiR-21 plus antimiR-10b was delivered via targeted and non-targeted PLGA-PEG nanoparticles. In the second cohort (batch ‘B’), we treated mice with various doses of TMZ after delivering antimiR-21 plus antimiR-10b via targeted and non-targeted PLGA-PEG nanoparticles. The treatments began when the tumor volume reached 25 mm^3^. For the purpose of analysis, we considered the tumor volume of 25 mm^3^ to be as 100% and presented the increase in tumor volume over time as increase in tumor volume percentage relative to Day 0. As shown in Figure 8C we found a significant decrease (*P* < 0.01) in tumor optical bioluminescence activity at day 11 in mice treated with 25 mg/kg TMZ, after antagomiRs were delivery by PLGA-PEG nanoparticles. The tumors treated with unmodified PLGA nanoparticles containing antagomiRs showed significantly (*P* < 0.01) reduced tumor size when compared to the cRGD-targeted PLGA-PEG nanoparticles. Interestingly the corresponding tumor volume data showed significant reduction in tumor volume starting on day 6 (Figure 8E), and this was maintained beyond that time point. In addition, a highly significant drop in tumor volume was observed at day 10 with unmodified PLGA-PEG nanoparticles containing antagomiRs, when compared with cRGD-targeted PLGA-PEG nanoparticles. Thus, unmodified PLGA nanoparticles were more effective in presensitizing tumors to TMZ (see Discussion). Figure 8D illustrates a significant (*P* < 0.01) decrease in bioluminescence activity in tumors that received cRGD-targeted PLGA-PEG nanoparticles along with 6.25 mg/kg of TMZ. Though the corresponding tumor volume that was measured on day 4 (Figure 8F) showed a greater reduction in tumor volume with a higher dose of TMZ (25 mg/kg), this difference was not significant on day 6 and beyond that time point, as all doses of TMZ caused a similar reduction in tumor size and bioluminescence signal by the end of the study, and all the doses showed high significance (*P* < 0.001) when compared with control (untreated group).

## DISCUSSION

The blood-brain barrier (BBB) presents a significant challenge to the delivery of therapeutic agents to the brain. Accordingly, most systemically administered drugs distribute throughout the body and do not reach the brain [20]. Trans-endothelial drug administration to overcome the BBB may be possible provided the physiological barrier of its component endothelia cells could be overcome [20]. Targeted delivery systems that recognize local receptors on the surface of endothelial or tumor cells are a promising strategy for drug delivery past the BBB. One approach in achieving this is through the use of ligand-guided, targeted nanoparticles. Brain tumors are highly angiogenic and overexpress cell adhesion receptors, such as integrin α_v_β_3_ that are also highly expressed on neovascular endothelial cells [24]. In a prior study using a mouse xenograft model of U87MG cells and magnetic resonance imaging, it was shown that cRGD-tagged PEGylated-copolymer-coated iron oxide nanoparticles successfully accumulated in tumors by initial targeting of α_v_β_3_ receptors [25].

The endogenous levels of miR-21 and miR-10b are elevated in GBM cells [12], and studies that have targeted these miRNAs using antisense oligonucleotides have disrupted the oncogenic properties of these cells [15, 26, 27]. To achieve delivery of these oligonucleotides, PLGA nanoparticles would have several advantages over other cationic nanoparticles. They are biocompatible, can encapsulate hydrophobic drugs and to a lesser extent hydrophilic drugs, allow sustained release of therapeutic molecules, and provide long-term stability [28–30]. PLGA is degraded in the cells by hydrolysis and eliminated through the Krebs cycle in the form of carbon dioxide and water. Hence it is considered biocompatible, with no toxicity associated with its use, and it is currently in clinical trials for various drug delivery applications [31, 32]. Nanoparticles prepared from PEG-PLGA polymer have shown long circulation time with minimal accumulation in the reticuloendothelial system (RES) and kidneys [33]. Indeed, previous cell culture studies have demonstrated non-targeted PLGA nanoparticles to be efficient nanocarriers for intracellular delivery and subsequent sustained release of antagomiR-21 and antagomiR-10b, leading to prolonged suppression of endogenous oncomiR functions in U87MG, Ln229, and T98G GBM cells in which baseline elevated expression of miR-21 and miR-10b was shown [15]. In this study, we functionalized PLGA nanoparticles with the ligand cRGD to target GBM xenografts *in vivo*. We first compared the efficiency of non-targeted PLGA-PEG and targeted cRGD-tagged PLGA-PEG nanoparticles, both constructed following a double emulsion solvent evaporation method. The size of the nanoparticles was <200 nm, similar to previously reported carriers, and they were highly monodispersed. However a reduction in the zeta potential was observed, that is, −15.3 ± 0.322 (targeted nanoparticles) and −17.1 ± 0.864 (non-targeted nanoparticles), which was less than previously reported studies (−26.3 ± 2.2) with unmodified PLGA nanoparticles [15]. The presence of a negative surface charge on nanoparticles is advantageous since it limits cellular uptake via nonspecific means and prevents nanoparticle agglomeration and adsorption of serum proteins. Moreover, both targeted and non-targeted nanoparticles showed high encapsulation efficiencies for antagomiRs (>90%) (Figure 2). We further investigated the cellular uptake of targeted and non-targeted PLGA nanoparticles in U87MG and Ln229 cells. Both nanoparticle types were tagged with Cy7.5 NIR dye to facilitate imaging their cellular uptake profile in culture using fluorescence microscopy. We found a significantly higher cellular uptake at 24 h in both U87MG and Ln229 cells for targeted nanoparticles when compared with non-targeted nanoparticles (Figure 3), most likely owing to receptor-mediated endocytosis. We further confirmed this early increase in the cellular uptake of targeted nanoparticles using FACS analysis, wherein the cRGD-tagged targeted nanoparticles showed uptake in a higher number of cells compared to cells treated with non-targeted nanoparticles. In addition, there was more uptake of targeted nanoparticles at early time points by U87MG cells mainly due to higher expression of integrin α_ν_β_3_ by U87MG cells compared to Ln229 cells. Thus, we here demonstrate the role of cRGD tagged peptide as a facilitator of receptor-mediated endocytosis of nanoparticles. By 48 h, the relative uptake increased for non-targeted nanoparticles owing to passive diffusion, resulting in a less significant difference in the relative fluorescence signal between targeted and non-targeted nanoparticles in cell culture.

Next, we performed a cell viability assay to determine the toxicity of targeted and non-targeted nanoparticles carrying 10 pmoles of antagomiR-21 and antagomiR-10b each, with and without increasing concentrations of TMZ (0 to 500 μM). The cell viability analysis was performed after 24 h and 48 h post-TMZ treatment, and we found that both targeted and non-targeted nanoparticles did not produce any significant toxicity on cells (Figure 4). However, a significant drop in cell viability was observed at a TMZ concentration of 62.75 μM and above. It was shown previously that pre-sensitizing GBM cells with antagomiR-21 and antagomiR-10b enhances the chemosensitivity of cells towards TMZ treatment (25% decrease in cell viability occurs at 500 μM TMZ concentration) [15]. As seen in our cell viability data, at 62.75 μM TMZ we observed approximately 20% decrease in cell viability in U87MG cells at 24 h and 40% at 48 h. This effect was more pronounced in Ln229 cells, with a >50% reduction in cell viability at 24 h. However, at 48 h the sustained anti-proliferation effect was observed only with TMZ at higher concentrations of 250 μM and 500 μM. Therefore, we here demonstrate that pre-sensitization of GBM cells with antagomiR-21 and antagomiR-10b may allow the possibility of using lower doses of concurrent TMZ to produce a comparable therapeutic effect. This result is in agreement with previous findings establishing that co-inhibition of miR-21 and miR-10b enhances the sensitivity of GBM cells to subsequent TMZ treatment. Although this study reveals no significant difference in cell viability after exposure to targeted or non-targeted nanoparticles, targeted nanoparticles show higher cellular uptake than non-targeted nanoparticles (Figure 3), thus prompting us to further analyze targeted nanoparticles plus TMZ co-treatment *in vivo*. TMZ has been shown to cause cell cycle arrest at G2/M phase by inducing DNA damage in human GBM cells [34]. As depicted in Figure 5, the live/dead assay performed using FACS analysis with cells stained with propidium iodide shows increased numbers of apoptotic cells after TMZ treatment. This is the case, especially when using targeted nanoparticles, followed by TMZ treatment for both U87MG and Ln229 cells. In addition, an increased cell accumulation at G2/M phase is observed in cells treated with targeted or non-targeted nanoparticles before TMZ treatment. However, there is no significant difference between the targeted and non-targeted nanoparticles when compared with the untreated control. Therefore co-inhibition of miR-21 and miR-10b enhances the sensitivity of GBM cells towards subsequent TMZ, but the significant antiproliferation effect occurs after TMZ treatment only.

To further validate the antiproliferative and chemosensitive effects of targeted and non-targeted nanoparticles plus the subsequent effects with TMZ treatment, we evaluated the downstream key targets for miR-21 and miR-10b, such as PTEN, PDCD4, HOXD10, and p53. Downregulation of miR-21 is known to enhance PTEN and PDCD4 tumor suppressor gene expression, and lead to decreased proliferation, increased apoptosis and decreased colony formation [35]. Increased expression of PTEN and PDCD4 is also known to increase the sensitivity of GBM cells to TMZ, as TMZ causes cell cycle arrest at the G2/M phase and inhibits the transition to G1 phase. HOXD10 has been reported as a direct target of miR-10b in human breast and esophageal cancers and its downregulation in GBM has been shown to affect cell invasion, tumor proliferation, and migration [15, 36, 37]. MiR10b is also pro-angiogenic; it represses the expression of HOXD10, which is known to exert anti-angiogenic effects. Thus, targeting miR-10b results in increased expression of HOXD10 [36]. In our study, owing to the low endogenous levels of miR-10b in cells, we do not see any significant differences in the relative gene expression of HOXD10. However, we find a significant increase in CASP3 expression for cells treated with targeted nanoparticles, with and without subsequent TMZ treatment. Caspase 3 protein is the most common cysteine protease initiating cellular breakdown during apoptosis.

We performed *in vivo* experiments using unmodified PLGA, non-targeted PLGA-PEG and cRGD-targeted PLGA-PEG nanoparticles carrying antagomiR-21 and antagomiR-10b. We had two different sets of animal experiments. In the first set (batch ‘A’) we evaluated the therapeutic efficacy of different nano-formulations in delivering antagomiRs to presensitize tumors towards subsequent TMZ treatment. In the second set (batch ‘B’) we evaluated the effects of different doses of TMZ on tumors after prior delivery of fixed quantities of antagomiRs through cRGD-targeted PLGA-PEG nanoparticles. We thus found that in the batch ‘A’ study, the use of unmodified PLGA nanoparticles resulted in the earlier therapeutic onset of tumor reduction in mice when compared to other groups. This may be owing to the higher negative charge of unmodified PLGA nanoparticles, which prevents adsorption of serum proteins [15] as compared to modified (non-targeted PLGA-PEG and cRGD-targeted PLGA-PEG) nanoparticles. Though in cell culture we had observed early higher cellular uptake of cRGD-targeted PLGA-PEG nanoparticles when compared with non-targeted PLGA-PEG nanoparticles, we did not observe a corollary of this *in vivo*. Although we did not perform *ex vivo* histological analyses of our xenograft tumors, we speculate that this observation may be owing to the already well described enhanced permeability of nanoparticles in the presence of abnormal tumor vasculature of xenografts, followed by enhanced retention in the absence of lymphatic vessels at the tumor site that might have facilitated equal efficiency for both targeted and non-targeted PLGA-PEG nanoparticles [38, 39]. Collectively this phenomenon is referred to as the ‘enhanced permeation and retention’ (EPR) effect, specifically for passive targeting based on nanoparticle size, as first described by Maeda et al [40]. Other studies have also demonstrated that the adsorption of serum proteins on PEG can modulate the stealth effect of PEGylated nanoparticles and destabilize them. In addition, the corona formed by serum proteins on PEGylated nanoparticles reduces their cellular uptake [41]. This effect was evident in our cellular uptake studies since the cRGD-targeted nanoparticles showed much higher cellular uptake signal when compared to the non-targeted nanoparticles in the presence of 2% FBS supplemented growth media, also confirming the cellular uptake by receptor-mediated endocytosis. By contrast, it has been reported that unmodified PLGA nanoparticles are rapidly internalized by cells either through fluid phase pinocytosis or clathrin-mediated endocytosis, following which, they escape the endo-lysosomes within 10 min to be released into the cytoplasm [42]. However, the use of PEG is preferred over unmodified nanoparticles for *in vivo* studies on account of it improving the systemic circulation of the nanoparticles thus modified [43]. This increased systemic circulation of nanoparticles enhances their biodistribution and bioavailability at the tumor site and diminishes aggregation (caused by serum proteins/opsonins) and subsequent uptake by the reticuloendothelial system [43]. For this reason we used cRGD-targeted PLGA-PEG nanoparticles in batch ‘B’ study. We reasoned here that cRGD-targeted nanoparticles would accumulate more on epithelium of neo-vasculature as well as cancer cell surfaces within tumors that are expressing α_ν_β_3_, compared to other tissues that are negative for this marker. However, we now question the need for PEGylating PLGA nanoparticles, since our batch ‘A’ animal study revealed an enhanced tumor suppression profile with unmodified PLGA nanoparticles (plus subsequent TMZ) when compared with targeted and non-targeted nanoparticles. Moreover, other studies have also indicated that modification of nanoparticles with PEG and other targeting moieties can hinder their effective endosomal release inside the cells [43], which can diminish the therapeutic efficacy of the nanoparticles. Also, although target specificity of nanoparticles was shown to be more effective in cell culture, we suggest that their use *in vivo* is of questionable value, at least as related to the treatment of subcutaneous xenografts that likely behave differently from intracranial tumors. We will further investigate these factors in future studies using more relevant orthotopic brain implant or genetically engineered models in mice. What is clear is that presensitizing tumors with antagomiR-21 and antagomiR-10b does make GBM xenografts chemosensitive to subsequent TMZ, resulting in tumor suppression at lower TMZ dosages.

## MATERIALS AND METHODS

### Ethics statement

Investigations have been conducted in accordance with the ethical standards according to the Declaration of Helsinki, according to national and international guidelines, and has been approved by the authors’ institutional review board.

### Materials

We obtained all chemical reagents of 95% purity and above from Sigma-Aldrich (St. Louis, MO, USA) and used them without further purification. Specifically, we procured acid-terminated poly (DL-lactide-coglycolide) (PLGA), lactide:glycolide 50:50 with an average molecular weight of 24,000-38,000 g/mol, polyvinyl alcohol of molecular weight 15,000 g/mol, 1-ethyl-3-(3- (dimethylamino)propyl)carbodiimide (EDC), N-hydroxysuccinimide (NHS) and diisopropylethylamine (DIPEA) from Sigma-Aldrich. We purchased MPEG-PLGA (5,000:90,000 DA, 50/50; LA/ GA) and PLGA-NH2 (Mn 10,000:35,000 Da) from PolySciTech (West Lafayette, Indiana), temozolomide (TMZ; molecular weight of 194.15 g/mol) from LKT Laboratories (St. Paul, MN, USA), cyclo (Arg-Gly-Asp-D-Phe-Lys) (cRGD) peptide from Peptide International, and PCI- 3661-PI and Cy7.5-NHS from Lumiprobe (Florida, USA). We obtained custom-synthesized phosphorothioate (PS) modified antagomiR-21 (UpCpApACAUCAGUCUGAUAAGpCpUpA) and antagomiR-10b (CpApCpAAAUUCGGUUCUACAGG pGpUpA) oligonucleotides with >90% purity from the protein and nucleic acid (PAN) facility at Stanford University.

### Synthesis of cRGD-functionalized PLGA

To obtain cRGD-functionalized PLGA polymer, we conjugated the PLGA-COOH polymer with NHS. Briefly, we dissolved PLGA-COOH (1 g) in 2 ml of dichloromethane (DCM), and added 27 mg of NHS and 46 mg of EDC to this solution. The reaction was allowed to take place at room temperature for 4 h. We precipitated the product with ethyl ether and washed thrice with ice cold mixture of ethyl ether and methanol (1:1) and vacuum dried overnight (at least for 6 h). To prepare PLGA-cRGD, we dissolved 100 mg of PLGA-NHS in 1 ml of DCM. We added 4.4 mg of cRGD to this solution, along with 2.8 mg of DIPEA. The reaction was allowed to proceed overnight (12 h). We precipitated the product using ice-cold methanol, followed by centrifugation at 5,000 rpm for 5 mins. The precipitate was washed thrice with methanol, vacuum dried overnight in the lyophilizer to obtain cRGD-conjugated PLGA polymer.

### Synthesis of Cy7.5 functionalized PLGA-PEG

We dissolved 125 mg of PLGA-NH2 in 1 ml of DCM and mixed the solution with 4.6 mg of Cy7.5-NHS and 2.8 mg of DIPEA. This reaction was allowed to take place overnight (12 h). After completion, we precipitated the product using ice-cold methanol, and washed thrice (5 ml each) with methanol by centrifugation at 5,000 rpm for 5 mins. We obtained the final product (Cy7.5-labeled PLGA) by vacuum drying overnight by lyophilization.

### Preparation of targeted and non-targeted PLGA-PEG nanoparticles encapsulating antagomiR-21 and antagomiR-10b

We prepared three types of nanoparticles (cRGD-targeted and non-targeted PLGA-PEG nanoparticles, and unmodified PLGA nanoparticles) encapsulating antagomiR-21 and antagomiR-10b following a double emulsion solvent evaporation method as published previously [9, 15, 17, 25]. Specifically, we prepared cRGD-targeted PLGA nanoparticles by mixing PLGA-PEG, PLGA-cRGD and PLGA-Cy7.5 in the ratio of 80:10:10 (w/w). Likewise, we prepared non-targeted nanoparticles by mixing PLGA-PEG and PLGA-Cy7.5 in the ratio of 90:10 (w/w), and unmodified PLGA nanoparticles were prepared using PLGA polymer alone, i.e. without PEG, Cy7.5 labelling or targeted peptide. We used unmodified PLGA nanoparticles for the *in vivo* studies only (see below). The physiochemical characterization and *in vitro* analysis of the unmodified PLGA nanoparticles have been detailed previously [17, 19]. To obtain nanoparticles, we first dissolved antagomiR-21 and antagomiR-10b in DNAse/RNAse free water. We then mixed 10 nmol of each of antagomiR-21 and antagomiR-10b to give a final volume of 50 μl in a microfuge tube. To this we further added 50 μl of spermidine (1 mg/ml), briefly vortexed, and incubated at room temperature for 15 min. We then added the antagomiRs-spermidine complex (100 μl) drop wise to 20 mg of PLGA (for unmodified nanoparticles) or PLGA-PEG, PLGA-cRGD and PLGA-Cy7.5 (for targeted nanoparticles), or PLGA-PEG and PLGA-Cy7.5 (for non-targeted nanoparticles) dissolved in 1 ml of DCM by stirring. We then sonicated this solution for 60 s at 40% amplitude in an ice bath. This resulted in a first emulsion, which was further added to 5 ml of 1% PVA solution drop-wise under constant stirring. We again emulsified the solution for 60 s at 60% amplitude in an ice bath. This resulted in a double-emulsified solution, which was stirred under reduced pressure for 4 h to evaporate the organic solvent. After 4 h, we collected the resulting nanoparticles by centrifugation at 3,500 rpm for 40 min using Amicon ultra-centrifugal filters (MW cut-off 100,000 Da). After centrifugation, we washed the nanoparticles twice with DNAse/RNAse free water to remove excess PVA and non-encapsulated antagomiRs. The nanoparticles thus obtained after washing were filtered through a 0.45 μm PVDF sterile filter unit to remove large aggregates. We then measured particle size, size distribution and surface charge (ζ-potential) using dynamic light scattering (DLS, Zetasizer Nano ZS, Malvern Instruments, UK). Additionally, we lyophilized the filtrate collected after washing the nanoparticles and analyzed it on a 3% agarose gel in Tris borate EDTA (TBE) buffer. We resolved the agarose gel at 90 V for 15 min to determine the antagomiR encapsulation efficiency of nanoparticles.

### Cell culture

We purchased the human GBM cell lines, U87MG (HTB-14) and Ln229 (CRL2611) from American Type Culture Collection (ATCC; VA), and used these within six months of purchase. ATCC's cell line authentication and characterization tests include checking for morphology using microscopy, growth curve analysis, isoenzymology for species verification, DNA fingerprinting for identity verification of human cell lines, and mycoplasma detection. We maintained the cells in Dulbecco's Modified Eagle Medium (DMEM; Corning Cellgro; VA) supplemented with 10% fetal bovine serum (FBS), penicillin-streptomycin (100 U/ml each) and incubated in a humidified chamber at 37° C with 5% CO_2_. For all cell culture experiments, we maintained the final concentration of antagomiRs at 10 pmoles/ml.

### Cellular uptake and cell cycle analysis

For cellular uptake analysis, we seeded U87MG and Ln229 cells at a density of 1 × 10^5^ cells/well in a complete growth medium with 10% FBS in 12-well plates. For cell cycle analysis, we seeded cells at a density of 1.5 × 10^5^ cells/well in 6-well plates. We treated the cells with cRGD-targeted and non-targeted PLGA nanoparticles containing antagomiR-21 and antagomiR-10b. We further treated these cells with 500 μM of TMZ for another 24 h. We studied the cellular uptake of nanoparticles at various time points using fluorescence microscopy (Olympus-IX81, Japan). We quantitatively analyzed the fluorescent images using ImageJ software. Similarly, we evaluated the fluorescence intensity of Cy7.5-nanoparticle uptake by FACS analysis in cells 24 h after treatment. For cell cycle status studies, we harvested both control and treated cells after the specified experimental conditions, and washed them with PBS. We fixed the cells in ice cold 70% ethanol at −20 °C overnight, and stained the cells with propidium iodide/RNase A/Triton X-100 (15 μg/ml / 10 μg/ml / 0.05%) for an additional 30 min. The cells were washed once with PBS and used for analysis using a FACS Aria III (BD Biosciences, CA) cell sorter. The results were analyzed using FlowJo FACS analysis software (Tree Star, OR).

### Cell viability assay

To perform cell viability in response to various treatment conditions, we seeded U87MG and Ln229 cells at a density of 5 × 10^3^/well in complete growth medium with 10% FBS in 96-well plates, the day prior to treatment. On the day of NP treatment, we washed the cells with PBS and supplemented them with DMEM containing 2% FBS. We then treated the cells with antagomiR-21 and antagomiR-10b encapsulated cRGD-targeted or non-targeted PLGA-PEG nanoparticles for a period of 24 h. After 24 h we treated the cells using various incremental concentrations of TMZ (0.98 μM to 500 μM) for additional periods of 24 h to 48 h. We measured the cell viability at 24 h and 48 h by treating cells with Resazurin (1% w/v stock) at a concentration of 0.02% (v/v) per well, followed by incubation in a humidified chamber at 37 °C with 5% CO_2_ for 2 h. We measured the cell viability using a multiwall plate reader (Infinite 1000, Tecan, Mannedorf, Switzerland) at excitation/emission wavelengths of 560/590 nm. The results were calculated and compared with control cells set at 100% viability.

### RNA extraction and qRT-PCR analysis

We used mirVana RNA extraction kits (Life Technologies, CA) for extracting total RNA from U87MG cells after various treatment conditions, following the manufacturer's protocol. We quantitated the total mRNA extracted using a Nanodrop 2000 spectrophotometer (Thermo scientific). We used 1 μg of total RNA to perform cDNA synthesis using a Quanta Biosciences reverse transcription kit (Beverley, MA) with the universal random primer as first strand synthesis primer. We performed real-time PCR using 5 μl of cDNA (50 ng of RNA equivalent) combined with TaqMan real-time PCR reagents for targets of miR-21 (PTEN and PDCD4) and miR-10b (HoxD10) and p53 in a total reaction volume of 20 μl. PCR parameters consisted of 2 min incubation at 50 °C, followed by activation of the Taq-DNA polymerase at 95 °C for 10 min and 60 cycles of 95 °C × 15 s, 60 °C × 60 s in an Eppendorf real-time PCR system. We normalized the expression of PTEN, PDCD4, HOXD10 and p53 to beta-actin housekeeping gene.

### Immunoblot assay

For immunoblot analysis, we washed the cells with PBS after different treatment conditions and lysed the cells in 100 μl of RIPA buffer containing protease inhibitor cocktail and 10 mM EDTA. We sonicated the cell lysates thoroughly to ensure the complete lysis of cells, and centrifuged at 16,000 g for 15 min at 4 °C. We collected the supernatant and measured the protein content using a Nanodrop 2000 spectrophotometer (Thermo scientific). We resolved 30 μg of protein in 4-12% gradient SDS/PAGE (Invitrogen) and electroblotted onto a 0.2 μm pore size nitrocellulose membrane (Schleicher & Schuell Biosciences, GmbH). We used SeeBlue® (Invitrogen, CA) protein marker to confirm the molecular mass and to test the complete transfer of protein to the membrane. We further blocked the membrane with 5% non-fat dry milk in TBS-T (TBS with 0.05% Tween 20) buffer for 1 h. We then incubated the membrane in 5 ml of fresh blocking solution and kept overnight with respective antibodies (rabbit mAb PTEN, rabbit mAb PDCD4, rabbit mAb HoxD10, rabbit mAb caspase-3, and mouse mAb-α-tubulin) obtained from Cell Signaling Technology (Danvers, MA) at 4 °C on a rotating platform. We washed the membrane thrice, 10 min each with PBS-T and incubated with HRP-conjugated goat anti-mouse/anti-rabbit secondary antibody respective to primary antibody for 2 h at room temperature. We washed the membrane another three times with PBS-T buffer before incubation with the chemiluminescent HRP substrate LumiGlo (Cell Signaling, MA), following the manufacturer's instructions. We detected the luminescence signal using an IVIS optical CCD camera (Caliper Life Sciences, Waltham, MA).

### *In vivo* tumor growth studies and bioluminescence imaging

We purchased 6-week-old nude mice (nu/nu), weighing 15-20 g from Charles River Laboratories (Wilmington, MA, USA) and housed them in an environment with controlled temperature (22 °C), humidity, and a 12 h light/dark cycle at our animal care facility. We conducted the animal experiments as per a protocol approved by our University Institutional Animal Care and Use Committee guidelines (APLAC-26748) and in adherence with the NIH Guide for the Care and Use of Laboratory Animals. We supplied standard mouse chow pellets (authoclaved) and water ad libitum. We established subcutaneous tumor xenografts of human U87MG cells engineered to stably express Fluc-eGFP reporter protein on both sides of the lower flanks of nude mice using one million cells per site in 100 μl solution (cell suspension in 50 μl PBS and 50 μl Matrigel-medium growth factor), and subsequently evaluated tumor volume (based on the formula: volume = [width^2^ × length/2]), and body weight of animals over time. We conducted these animal studies in two different batches. Batch ‘A’ had four different treatment conditions, with five animals in each group. All treatment groups received 100 μl of nanoparticle solution containing 280 pmoles of antagomiR-21 and antagomiR-10b (each in their respective nanoparticles) via tail vein injections, followed by 25 mg/kg of TMZ via intraperitoneal injections. We performed a pre-scan on tumors to measure the bioluminescence signal a day prior (Day 0) to nanoparticle injection. We injected the nanoparticles at 7 days post tumor cell injection when the tumor reached 25 mm^3^. The TMZ was administered one day after nanoparticle injection, for two consecutive days. We repeated this protocol of administering nanoparticles followed by TMZ treatment once every 3 days. The treatment groups within batch ‘A’ were: 1) Untreated control, 2) Unmodified PLGA nanoparticles injection, 3) Non-targeted PLGA-PEG nanoparticles injection, and 4) cRGD-targeted PLGA-PEG nanoparticles injection. Similarly, we planned another group of animals for treatment as Batch ‘B’, where we analyzed the cumulative effects of administering cRGD-targeted PLGA-PEG nanoparticles containing antagomiR-21 and antagomiR-10b, and the chemosensitivity (diminished growth) of these tumors after administering different doses of TMZ. Batch ‘B’ had four different treatment groups, with three animals bearing two tumors each (n=6), in each group. All animals were divided into separate treatment groups: 1) Untreated control, 2) 6.25 mg/kg TMZ, 3) 12.5 mg/kg TMZ, and 4) 25 mg/kg TMZ. To image FLuc, we intraperitoneally injected the animals with 3 mg of its substrate D-Luciferin in 100 μL PBS, 5 min before signal acquisition. For bioluminescence imaging, we imaged the animals with a LagoX instrument (from Spectral Instruments, LLS, Tucson, AZ) by integrating emitted photons for a period of 1 min for 20 acquisitions. We analyzed the images using AMIVIEW software provided by Spectral Instruments. To quantify the number of emitted photons, we drew regions of interest over the areas of FLuc signals, and recorded the maximum photons per second per square centimeter per steradian (p/sec/cm^2^/sr) generated using AMIVIEW software.

### Statistical analysis

We performed statistical analysis using Students *t* test. We presented the data as mean ± SEM, and mean ± SD for qRT-PCR analysis. We considered the significance levels at (^*^) *P* ≤ 0.05, (^**^) *P* ≤ 0.01, and (^***^) *P* ≤ 0.001.

## CONCLUSIONS

We developed cRGD-targeted and non-targeted nanoparticles with optimized loading for delivering antagomiR-21 and antagomiR-10b to GBM cells. In cell culture, the cRGD-targeted PLGA-PEG nanoparticles show enhanced antagomiR encapsulation efficiency and improved cellular uptake in U87MG and Ln229 cells when compared with non-targeted PLGA-PEG nanoparticles. When tested in cell viability and apoptotic assays these nano-formulations show efficient anticancer effects at lower doses of TMZ treatment. Further quantification of cell cycle arrest after TMZ treatment of cells pre-exposed to nano-formulations confirms an increased accumulation of cells at the G2/M phase. The qRT-PCR and immunoblot assays performed on the key targets of miR-21 and miR-10b reveal increased expression of PTEN, PDCD4, and CASP3 in U87MG cells treated with targeted nanoparticles, when compared with non-targeted nanoparticles. On the other hand, *in vivo* systemic administration of nanoparticles leads to an EPR effect in mouse subcutaneous xenografts, and, regardless of surface modification, all animals treated with different nano-formulations show a decrease in tumor volume. However, this decrease is significantly more for the unmodified control PLGA nanoparticles. In addition, mice treated with targeted nanoparticles show an efficient therapeutic response for all TMZ doses tested in this study, demonstrating that the use of a lower dosage of TMZ (in conjunction with therapeutic miRNAs) can equally and efficiently result in reducing tumor volumes. The encouraging results of this study establish the foundation for our use of therapeutic miRNAs in many more future *in vivo* investigations using orthotopic mouse brain models of GBM, especially when accompanied by novel strategies to bypass the BBB. Once translated clinically in the future, we anticipate that presensitizing tumors with nanoparticle-delivered therapeutic antagomiRs could potentially represent a useful clinical strategy to lessen unwanted side effects of TMZ treatment of GBMs.
